# Correction to “Prognostic Value of Intrarenal Venous Flow Analysis Using Pulsed‐Wave Doppler”

**DOI:** 10.1111/jvim.70266

**Published:** 2025-11-06

T. Morita, H. Terukina, and M. Yamasaki, “Prognostic Value of Intrarenal Venous Flow Analysis Using Pulsed‐Wave Doppler,” *Journal of Veterinary Internal Medicine* 39 (2025): e70260. https://doi.org/10.1111/jvim.70260.

In the above‐mentioned article, the title of the article is incorrect and needs to be changed as below:

Prognostic Value of Intrarenal Venous Flow Analysis Using Pulsed‐Wave Doppler Ultrasonography in Dogs with Myxomatous Mitral Valve Disease

The original article has also been corrected.

We apologize for this error.

